# LCZ696, a promising novel agent in treating hypertension (a meta-analysis of randomized controlled trials)

**DOI:** 10.18632/oncotarget.22442

**Published:** 2017-11-14

**Authors:** Liwen Ye, Jian Wang, Qingwei Chen, Xixi Yang

**Affiliations:** ^1^ Department of Geriatric Cardiology, The Second Affiliated Hospital of Chongqing Medical University, Chongqing, 400010, China

**Keywords:** blood pressure, cardiovascular, cardiovascular disease

## Abstract

**Background:**

To determine the effectiveness and safety of LCZ696 for the clinical treatment of hypertension, we performed a meta-analysis of the previous clinical trials.

**Methods:**

Relevant English articles and randomized controlled trials were searched in Pubmed, Embase, EBSCO, Cochrane base and ClinicalTrials.gov. The last search date was July 20th, 2017.

**Results:**

Compared with 20mg olmesartan, 200mg and 400mg LCZ696 outperformed olmesartan in terms of reducing mean sitting systolic blood pressure, mean ambulatory systolic blood pressure, mean sitting diastolic blood pressure and mean ambulatory diastolic blood pressure. Compared with 20mg olmesartan, 200mg and 400mg LCZ696 was better than olmesartan in terms of reducing mean sitting pulse pressure. And these studies showed that 400mg LCZ696 was better than 20mg olmesartan in terms of reducing mean ambulatory pulse pressure, however, there was no significant difference between 200mg LCZ696 and 20mg olmesartan in terms of redducing mean ambulatory pulse pressure. In addition, 200mg and 400mg LCZ696 was better than placebo in terms of reducing blood pressure parameters mentioned above. Compared with placebo or 20 mg olmesartan, LCZ696 showed no superiority in terms of reducing adverse events or serious adverse events.

**Conclusions:**

LCZ696 at 200 mg or 400 mg was better at reducing most of blood pressure parameters than 20 mg olmesartan or placebo. Compared with placebo or 20 mg olmesartan, 200 mg or 400 mg LCZ696 do not result in more adverse events in treating hypertension.

## INTRODUCTION

Hypertension is one of the most common cardiovascular diseases [1]. Although it can be treated with a variety of drugs, there is ongoing and intensive research to develop drugs that reduce blood pressure (BP) more effectively.

A potential antihypertensive drug target that has received considerable interest over the years is neprilysin. This endopeptidase is responsible for the degradation of natriuretic peptides (NPs), which are hormones with natriuretic and kaliuretic properties that are mainly secreted by the heart [2]. The NPs include atrial NP (ANP), brain/B-type NP (BNP), and C-type NP (CNP). Since NPs can increase natriuresis and diuresis, they can lower BP. They also directly regulate the renin-angiotensin-aldosterone system (RAAS): they can reduce renin release, which in turn decreases the secretion of angiotensin II and aldosterone [3]. However, despite this direct effect of NPs on the RAAS, several studies have shown that when the RAAS is inhibited by targeting other RAAS-regulating mechanisms, concomitant neprilysin inhibition has a greater effect on BP than when either target is inhibited alone [4–6]. For example, although the neprilysin inhibitors ecadotril, racecadotril, and candoxatril fail to treat heart disease and hypertension effectively [7–9], many clinical studies have shown that omapatrilat, which inhibits various proteases including neprilysin, angiotensin-converting enzyme (ACE), and aminopeptidase, effectively treats hypertension. Therefore, such combined therapy may be a potential way to treat hypertension.

Unfortunately, omapatrilat has a number of side effects, including an elevated risk of angioedema, which have restricted its application [10, 11]. Consequently, at present, a number of other drugs that can inhibit both neprilysin and the RAAS are under development or are being tested.

One of these is LCZ696, which is a novel angiotensin receptor-neprilysin inhibitor (ARNI). A clinical trial testing the efficacy of LCZ696 for hypertension found that this drug does not increase the risk of angioedema [12]. Many clinical trials have been carried out to test the effectiveness of LCZ696 for the treatment of hypertension, but only a few have been large-scale clinical trials. To determine the effectiveness and safety of LCZ696 for the clinical treatment of hypertension, we performed a meta-analysis of the previous clinical trials.

## RESULTS

### Characteristics of the selected studies

**Figure 1:**
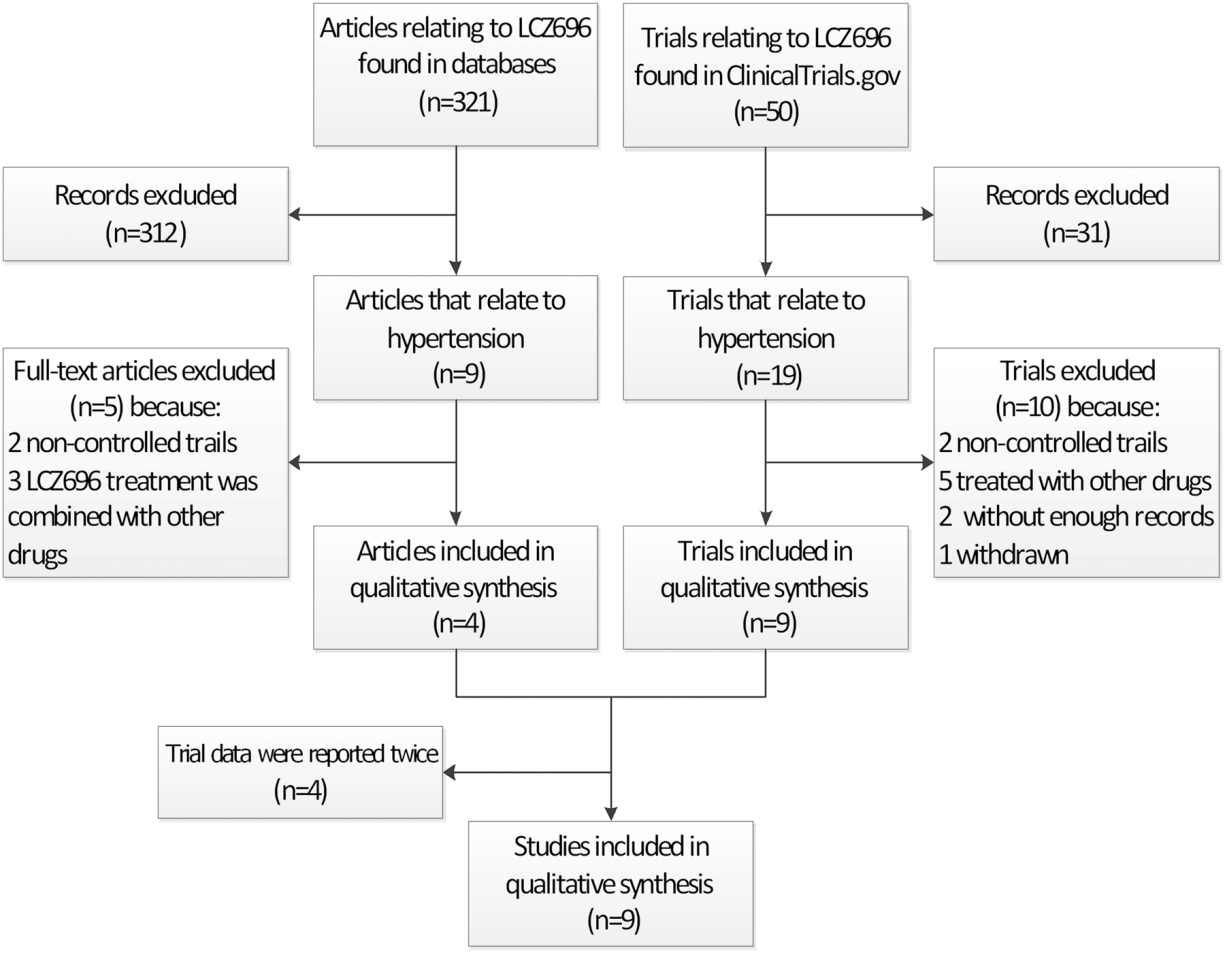
The number of studies that were screened, assessed for eligibility, and included in the meta-analysis

Figure 1 shows the flow diagram (Figure 1) of the meta-analysis, which is required by the PRISMA Statement. In total, 321 papers that related to LCZ696 were found in PubMed, EMBASE, and the Cochrane database. Of these, nine related to the treatment of hypertension. Of those eight papers, two reported uncontrolled trials and in three the experimental group patients were treated with amlodipine as well as LCZ696. Only four RCTs in the remaining four papers met all of the eligibility criteria [12, 14, 15, 18]. In addition, 50 registered trials that related to LCZ696 were found in the ClinicalTrials.gov website. Of these, 19 related to the treatment of hypertension. Of those 19 papers, two were not controlled trials and in five the experimental group patients were treated with amlodipine as well as LCZ696. Moreover, two lacked sufficient records and one was withdrawn. The remaining nine trials were RCTs that conformed to the inclusion criteria. The data of four of these nine RCTs (NCT00549770, NCT01193101, NCT01281306 and NCT01692301) were published in the four articles that were selected for meta-analysis after searching the medical databases, as described above [12, 14, 15, 18]. Thus, a total of nine RCTs were selected for this meta-analysis. These RCTs included 5130 patients, of whom 3173 were in the LCZ696 group, 1634 in the olmesartan group, and 323 in the placebo group. The general characteristics of the patients in these RCTs are shown in Table 1.

**Table 1:** Characteristics of the included trials

ClinicalTrials.gov identifier	Drug dose(mg)		Patients(n)		Gender(Female)		Age(Years)		Follow-uptime (Weeks)
	LCZ696(mg)	Control	LCZ696	Control	LCZ696	Control	LCZ696	Control	
NCT00549770[14]	200	Placebo	169	173	77	79	54±9.7	54±10.6	8
	400		172		75		52±10.9		
NCT01193101[12]	200	Placebo	101	92	27	24	52.1±8.82	50.9±10.65	8
	400		96		23		50.9±9.81		
NCT01281306[18]	400	Placebo	142	58	71	29	61.2±10.6	60.8±11.81	8
NCT01599104	200	Olmesartan 20mg	387	389	123	103	57.9±10.87	59.6±10.50	8
	400		385		117		58.7±10.50		
NCT01615198	200	Olmesartan 20mg	296	292	154	140	70.5±4.67	70.9±4.67	10
NCT01692301[15]	200	Olmesartan 20mg	229	225	110	107	68.2±5.73	67.2±5.79	52
NCT01785472	200	Olmesartan 20mg	479	484	227	223	57.5±10.17	57.4±10.14	8
	400		472		229		58.1±9.71		
NCT01870739	400	Olmesartan 20mg	57	57	20	17	60.5±7.8	59.2±13.1	52
NCT01876368	200	Olmesartan 20mg	188	187	91	92	57.1±10.19	58.0±9.09	8

U: Unclear; -: Null.

### Risk of bias

**Figure 2:**
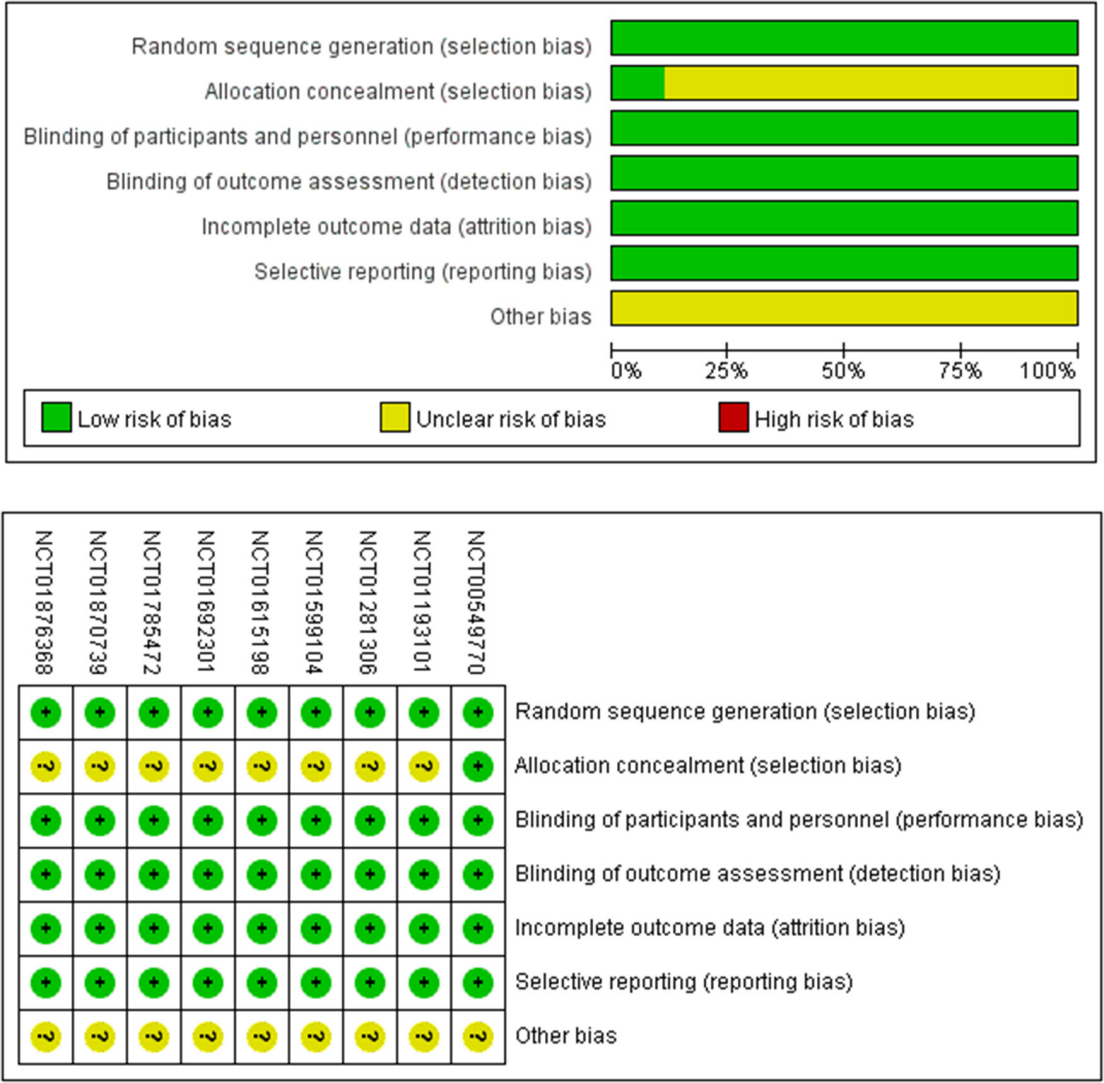
Methodological quality assessment

**Figure 3:**
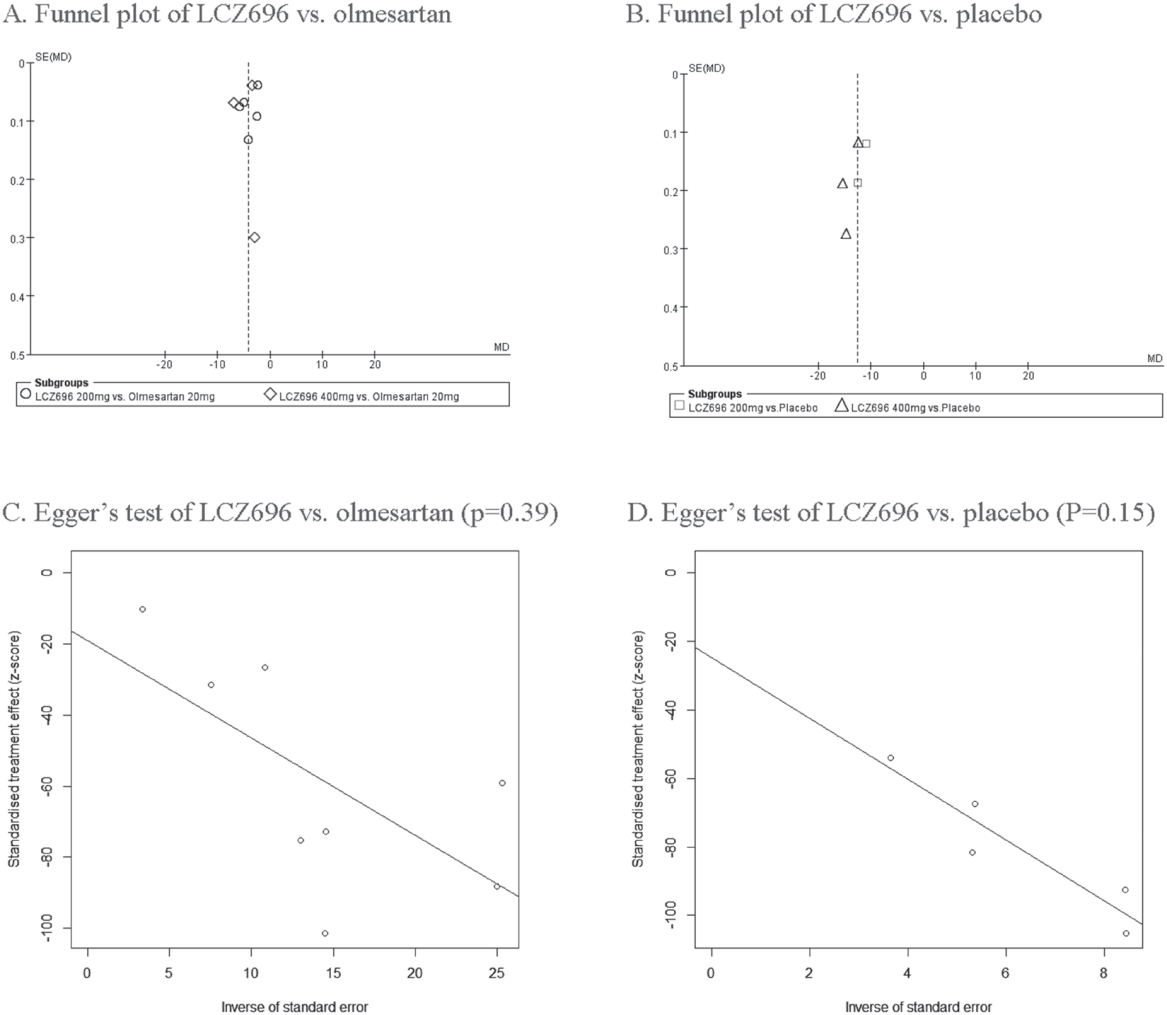
Funnel plot of LCZ696 vs. olmesartan **(A)** and placebo **(B)**. Egger's test of LCZ696 vs. olmesartan **(C)** and placebo **(D)**.

The quality of this study was assessed by using the Cochrane Collaboration bias risk tools (Figure 2). All nine RCTs were randomized controlled double-blinded clinical trials. The funnel plot indicates symmetry, which indicates that there is little publication bias (Figure 3A, 3B). Moreover, the outcome of Eegger's test also supports this conclusion (LCZ696 vs. olmesartan, p=0.39; LCZ696 vs. placebo, p=0.15) (Figure 3C, 3D).

### Indicators of LCZ696 effectiveness

#### msSBP

All nine RCTs reported msSBP. Due to high heterogeneity (p<0.01, I^2^=96.6%), the random effects model of DerSimonian and Laird was used to analyze the data.

**Figure 4:**
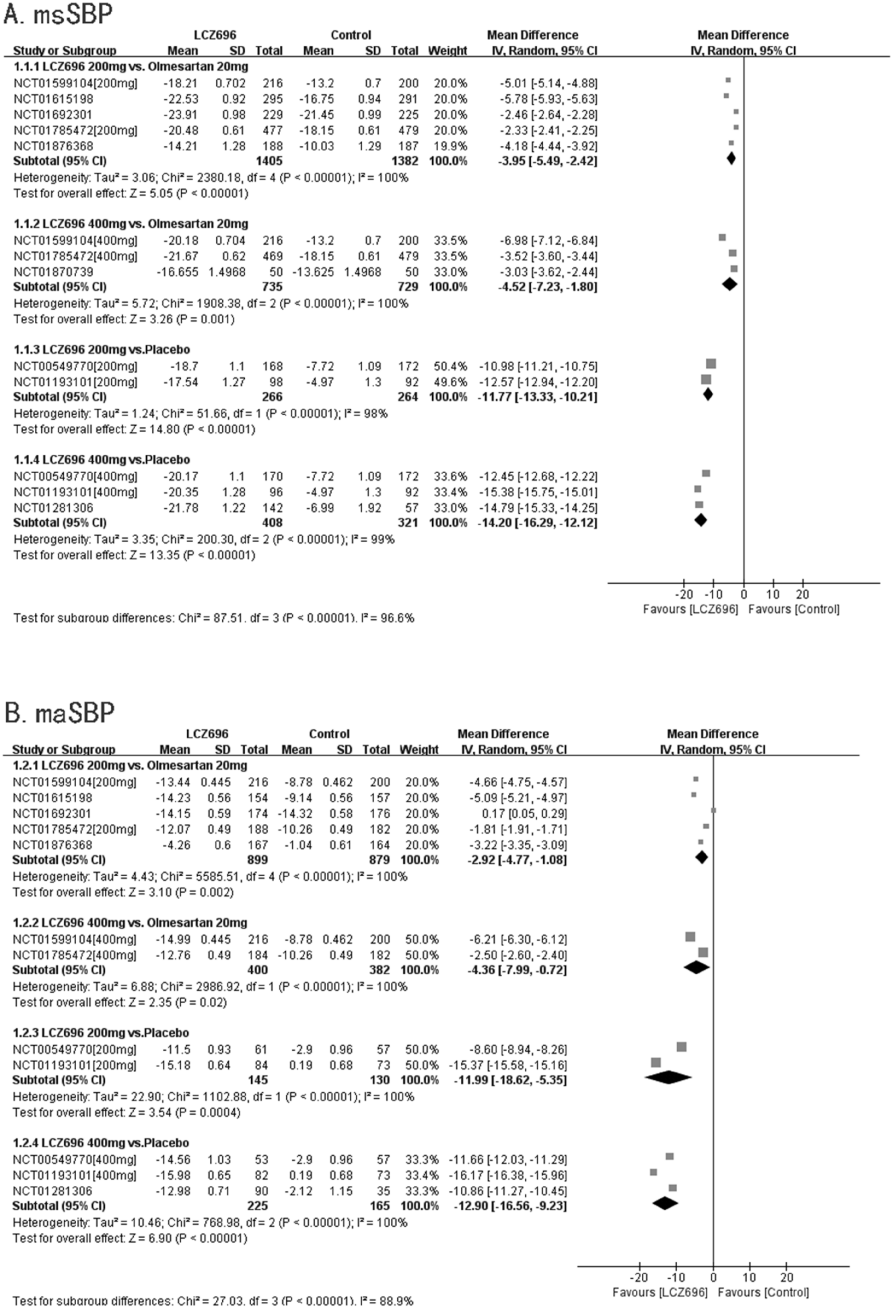
Forest plot of msSBP **(A)** and maSBP **(B)**. The comparisons of LCZ696 with a control group were classified according to the dosage of LCZ696 and whether the control group was treated with olmesartan or placebo. (200 mg: treatment with LCZ696 at 200 mg daily; 400 mg: treatment with LCZ696 at 400mg daily).

Five RCTs compared the effectiveness of 200 mg LCZ696 with that of 20 mg olmesartan. In these studies, LCZ696 was better than olmesartan in terms of reducing msSBP (200 mg LCZ696 *vs*. 20 mg olmesartan, WMD=-3.95, 95% CI: -5.49, -2.42; p<0.01). Three RCTs compared the effectiveness of 400 mg LCZ696 with that of 20 mg olmesartan. In these trials, LCZ696 was better than olmesartan in terms of reducing msSBP (400 mg LCZ696 *vs*. 20 mg olmesartan, WMD=-4.52, 95% CI: -7.23, -1.80; p<0.01). Two RCTs compared the effectiveness of 200 mg LCZ696 with placebo. In these studies, LCZ696 was also better than placebo in terms of reducing msSBP (200 mg LCZ696 *vs*. placebo, WMD=-11.77, 95% CI: -13.33, -10.21; p<0.01). Three RCTs compared the effectiveness of 400 mg LCZ696 with that of placebo. In these trials, LCZ696 was better than olmesartan in terms of reducing msSBP (400 mg LCZ696 *vs*. placebo, WMD=-14.20, 95% CI: -16.29, -12.12; p<0.01). (Figure 4A).

#### maSBP

Eight RCTs reported maSBP. Five RCTs compared the effectiveness of 200 mg LCZ696 with that of 20 mg olmesartan. In these trials, LCZ696 was better than olmesartan in terms of reducing maSBP (200 mg LCZ696 *vs*. 20mg of olmesartan, WMD=-2.92, 95% CI: -4.77, -1.08; p<0.01). Two RCTs compared the effectiveness of 400 mg LCZ696 with that of 20 mg olmesartan. These studies showed that LCZ696 was better than olmesartan in terms of reducing maSBP (400mg LCZ696 *vs*. 20 mg olmesartan, WMD=-4.36, 95% CI: -7.99, -0.72; p=0.02). Two RCTs compared the effectiveness of 200 mg LCZ696 with that of placebo. In these trials, LCZ696 was better than placebo in terms of reducing maSBP (200 mg LCZ696 *vs*. placebo, WMD=-11.99 95% CI: 18.62, -5.35; p<0.01). Three RCTs compared the effectiveness of 400 mg LCZ696 with that of placebo. These studies showed that LCZ696 was better than placebo in terms of reducing maSBP (400 mg LCZ696 *vs*. placebo, WMD=-12.90, 95% CI: -16.56, -9.23; p<0.01). (Figure 4B).

#### msDBP

**Figure 5:**
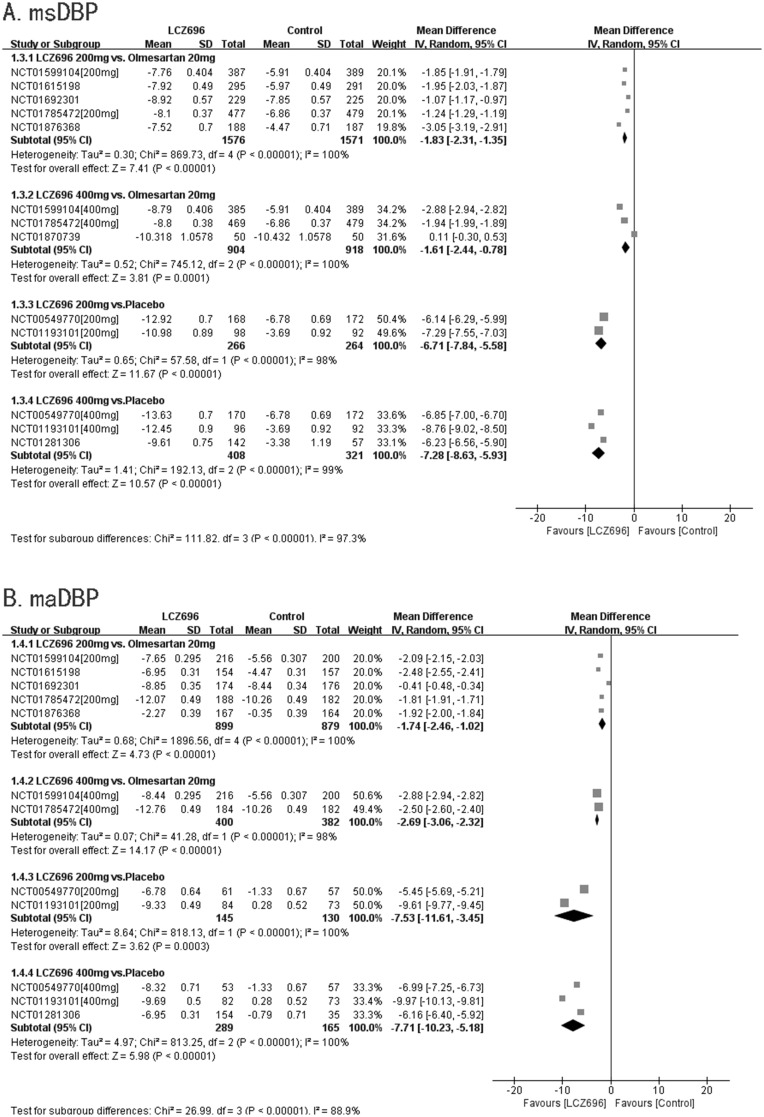
Forest plot of msDBP **(A)** and maDBP **(B)**. The comparisons of LCZ696 with a control group were classified according to the dosage of LCZ696 and whether the control group was treated with olmesartan or placebo.

Nine RCTs reported msDBPs. Five RCTs compared the effectiveness of 200 mg LCZ696 with that of 20 mg olmesartan. These studies showed that LCZ696 was better than olmesartan in terms of reducing msDBP (200 mg LCZ696 *vs*. 20 mg olmesartan, WMD=-1.83, 95% CI: -2.31, -1.35; p<0.01). Three RCTs compared the effectiveness of 400 mg LCZ696 with that of 20 mg olmesartan. These trials showed that LCZ696 was better than olmesartan in terms of reducing msDBP (400 mg LCZ696 *vs*. 20 mg olmesartan, WMD=-1.61, 95% CI: -2.44, -0.78; p<0.01). Two RCTs compared the effectiveness of 200 mg LCZ696 with that of placebo. These studies showed that LCZ696 was better than placebo in terms of reducing msDBP (200 mg LCZ696 *vs*. placebo, WMD=-6.71, 95% CI: -7.84, -5.58; p<0.01). Three RCTs compared the effectiveness 400 mg LCZ696 with that of placebo. These trials showed that LCZ696 was better than placebo in terms of reducing msDBP (400 mg LCZ696 *vs*. placebo, WMD=-7.28, 95% CI: -8.63, -5.93; p<0.01). (Figure 5A).

#### maDBP

Eight RCTs reported maDBP. Five RCTs compared the effectiveness of 200 mg LCZ696 with that of 20mg olmesartan. These trials showed that LCZ696 is better than olmesartan in terms of reducing maDBP (200 mg LCZ696 *vs*. 20 mg olmesartan, WMD=-1.74, 95% CI: -2.46, -1.02; p<0.01). Two RCTs compared the effectiveness of 400 mg LCZ696 with that of 20 mg olmesartan. These studies showed that LCZ696 was better than olmesartan in terms of reducing maDBP (400 mg LCZ696 *vs*. 20 mg olmesartan, WMD=-2.69, 95% CI: -3.06, -2.32; p<0.01). Two RCTs compared the effectiveness of 200 mg LCZ696 with that of placebo. These trials showed that LCZ696 is better than olmesartan in terms of reducing maDBP (200 mg LCZ696 *vs*. placebo, WMD=-7.53, 95% CI: -11.61, -3.45; p<0.01). Three RCTs compared the effectiveness of 400 mg LCZ696 with that of placebo. These studies showed that LCZ696 was better than placebo in terms of reducing maDBP (400 mg LCZ696 *vs*. placebo, WMD=-7.71, 95% CI: -10.23, -5.18; p<0.01). (Figure 5B).

#### msPP

**Figure 6:**
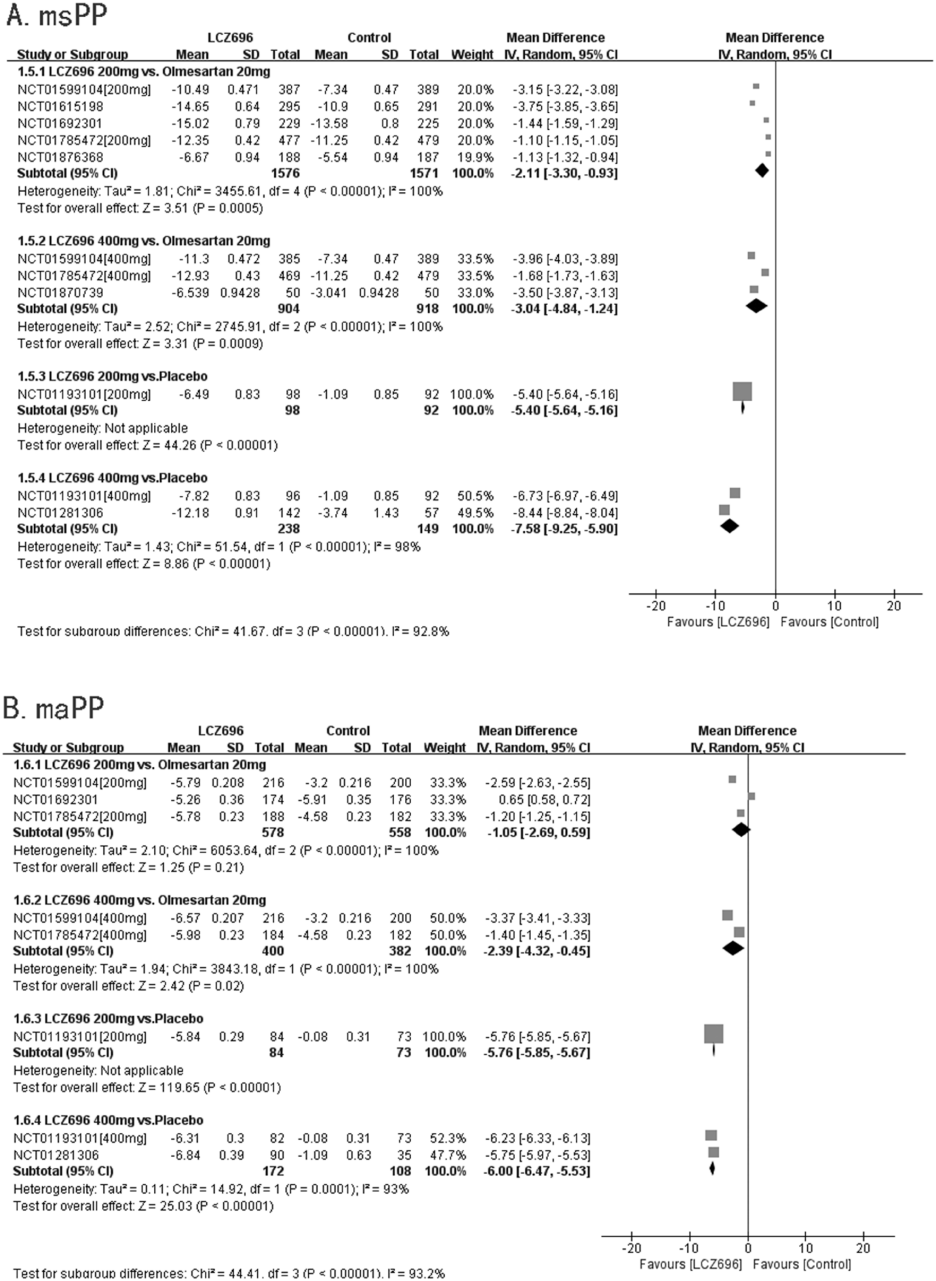
Forest plot of msPP **(A)** and maPP **(B)**. The comparisons of LCZ696 with a control group were classified according to the dosage of LCZ696 and whether the control group was treated with olmesartan or placebo.

Eight RCTs reported msPP. Five RCTs compared the effectiveness of 200 mg LCZ696 with that of 20 mg olmesartan. These studies showed that LCZ696 was better than olmesartan in terms of reducing msPP (200 mg LCZ696 *vs*. 20 mg olmesartan, WMD=-2.11, 95% CI: -3.30, -0.93; p<0.01). Three RCTs compared the effectiveness of 400 mg LCZ696 with that of 20 mg olmesartan. These trials showed that LCZ696 was better than olmesartan in terms of reducing msPP (400 mg LCZ696 *vs*. 20 mg olmesartan, WMD=-3.04, 95% CI: -4.84, -1.24; p<0.01). One RCT compared the effectiveness of 200 mg LCZ696 with that of placebo. This study showed that LCZ696 was better than placebo in terms of reducing msPP (200 mg LCZ696 *vs*. placebo, WMD=-5.40, 95% CI: -5.64, -5.16; p<0.01). Two RCTs compared the effectiveness of 400 mg LCZ696 with that of 20 mg olmesartan. These trials showed that LCZ696 was better than placebo in terms of reducing msPP (400 mg LCZ696 *vs*. placebo, WMD=-7.58, 95% CI: -9.25, -5.90; p<0.01).(Figure 6A).

#### maPP

Five RCTs reported maPPs. These three RCTs compared the effectiveness of 200 mg LCZ696 with that of 20 mg olmesartan. However, the result showed that there was no significant difference between 200 mg LCZ696 and 20 mg olmesartan in terms of reducing maPP (200 mg LCZ696 *vs*. 20 mg olmesartan, WMD=-1.05, 95% CI: -2.69, -0.59; p=0.21). Two RCTs compared the effectiveness of 400 mg LCZ696 with that of 20 mg olmesartan. These studies showed that LCZ696 was better than olmesartan in terms of reducing maPP (400 mg LCZ696 *vs*. 20 mg olmesartan, WMD=-2.39, 95% CI: -4.32, -0.45; p=0.02). One RCT compared the effectiveness of 200 mg LCZ696 with that of placebo. This trial showed that LCZ696 was better than placebo in terms of reducing maPP (200 mg LCZ696 *vs*. placebo, WMD=5.76, 95% CI: -5.85, -5.67; p<0.01). Two RCTs compared the effectiveness of 400 mg LCZ696 with that of placebo. These studies showed that LCZ696 was better than olmesartan in terms of reducing maPP (400 mg LCZ696 *vs*. placebo, WMD=-6.00, 95% CI: -6.47, -5.53; p<0.01). (Figure 6B).

### Safety

#### AEs

**Figure 7:**
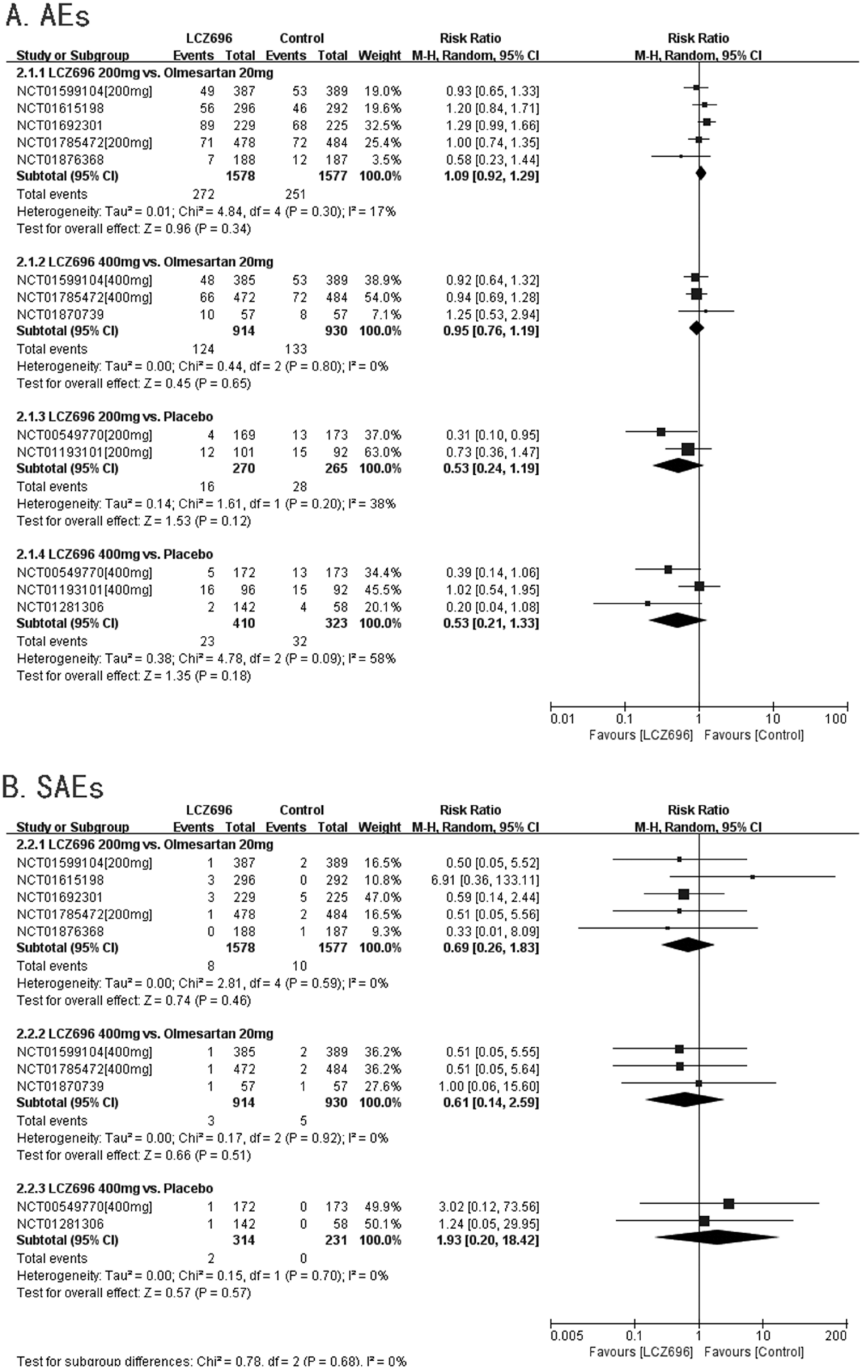
Forest plot of Aes **(A)** and SAEs **(B)**. The comparisons of LCZ696 with a control group were classified according to the dosage of LCZ696 and whether the control group was treated with olmesartan or placebo.

Nine RCTs had reported AEs. Five RCTs compared the safety of 200 mg LCZ696 with that of 20 mg olmesartan. The result showed that there was no significant difference between 200 mg LCZ696 and 20 mg olmesartan in terms of reducing AEs (200 mg LCZ696 *vs*. 20 mg olmesartan, RR=1.09, 95% CI: 0.92, 1.29; p=0.34). Three RCTs compared the safety of 400 mg LCZ696 with that of 20 mg olmesartan. The result showed that there was no significant difference between 400 mg LCZ696 and 20 mg olmesartan in terms of reducing AEs (400 mg LCZ696 *vs*. 20 mg olmesartan, RR=0.95, 95% CI: 0.76, 1.19; p=0.65). Two RCTs compared the safety of 200 mg LCZ696 with that of placebo. The result showed that there was no significant difference between 200 mg LCZ696 and placebo in terms of reducing AEs (200 mg LCZ696 vs. placebo, RR=0.53, 95% CI: 0.24, 1.19; p=0.12). Three RCTs compared the safety of 400 mg LCZ696 with that of placebo. The result showed that there was no significant difference between 400 mg LCZ696 and placebo in terms of reducing AEs (400 mg LCZ696 vs. placebo, RR=0.53, 95% CI: 0.21, 1.33; p=0.18). (Figure 7A).

#### SAEs

Eight RCTs had reported SAEs. Three RCTs compared the safety of 200 mg LCZ696 with that of 20 mg olmesartan. The result showed that there was no significant difference between 200 mg LCZ696 and 20 mg olmesartan in terms of reducing SAEs (200 mg LCZ696 *vs*. 20 mg olmesartan, RR=0.69, 95% CI: 0.26, 1.83; p=0.46). Three RCTs compared the safety of 400 mg LCZ696 with that of 20 mg olmesartan. The result showed that there was no significant difference between 400 mg LCZ696 and 20 mg olmesartan in terms of reducing SAEs (400 mg LCZ696 *vs*. 20 mg olmesartan, RR=0.61, 95% CI: 0.14, 2.59; p=0.51). Two RCTs compared the safety of 400 mg LCZ696 with that of placebo. The result showed that there was no significant difference between 400 mg LCZ696 and placebo in terms of reducing SAEs (400 mg LCZ696 vs. placebo, RR=1.93, 95% CI: 0.20, 18.42; p=0.57). (Figure 7B).

## DISCUSSION

Water retention due to high sodium levels and particularly the activation of the RAAS are considered to be the main reasons for hypertension. When RAAS is activated, angiotensin II binds to angiotensin II receptor type 1, which promotes vascular smooth muscle contraction, increases aldosterone synthesis and secretion, and increases arterial smooth muscle cell growth and proliferation. This ultimately increases BP. ARBs inhibit the binding of angiotensin II to angiotensin II receptor type 1, thereby inhibiting the activation of RAAS. This in turn suppresses vascular smooth muscle contraction and aldosterone secretion. This reduces vascular peripheral resistance, thereby lowering BP [19].

NPs are a family of hormones that maintain the sodium-water balance. Nishikimi *et al*.[20] showed that intravenous infusion of NPs in mice reduces the secretion of renin by renal glomerular cells. This reduces the formation of plasma angiotensin II and aldosterone, and thereby indirectly promotes vascular smooth muscle relaxation. Since NPs are degraded by the zinc-containing metallopeptidase neprilysin [21], inhibiting the activity of neprilysin may be a novel way to treat hypertension.

LCZ696 is a novel dual-acting ARNI that consists of a 1:1 mixture of the valsartan and sacubitril, which is the pro-drug of the active neprilysin inhibitor LBQ657. After administration, valtarsan is released and sacubitril is rapidly hydrolyzed by specific esterases into LBQ657 [22, 23]. Valsartan, being an ARB, blocks RAAS activation, thereby eventually decreasing BP. By contrast, LBQ657 blocks the NP-degrading activity of neprilysin, thereby elevating NP levels and restoring the ideal sodium-water balance. This eventually contributes to the control of BP [24].

The present meta-analysis showed that compared with both placebo and 20 mg olmesartan, LCZ696 significantly reduced msSBP, maSBP, msDBP, and maDBP. The fact that LCZ696 reduced BP compared with placebo indicates that LCZ696 can lower BP effectively. The fact that LCZ696 was more effective in reducing BP than 20 mg olmesartan indicates that either LCZ696 is more effective than 20 mg olmesartan or the dosage of olmesartan in the studies was insufficient. In this paper, 200 mg and 400 mg LCZ696 outperformed 20 mg olmesartan in reducing BP. If the dosage of olmesartan is increased, what effect would this have on the outcome? The study NCT00549770 employing 100 mg LCZ696 vs. 80 mg olmesartan, 200 mg LCZ696 vs. 160 mg olmesartan, and 400 mg LCZ696 vs. 320 mg olmesartan showed that the effect of LCZ696 in decreasing msSBP, maSBP, msDBP, and maDBP was better than that achieved by olmesartan. In the NCT01281306 study, the results showed that LCZ696 was better at decreasing msSBP, maSBP, msDBP, maDBP, msPP, and maPP than valsartan when the effectiveness of 400 mg LCZ696 and 320 mg valsartan were compared. At present, only a few studies have reported changes in the control groups with different drug dosages; thus, further large-scale clinical trials using different drug dosages in the control group are required.

This meta-analysis also showed that compared with placebo, 200 mg and 400 mg LCZ696 significantly reduced msPP and maPP. Compared with 20 mg olmesartan, 400 mg LCZ696 was more effective in decreasing msPP and maPP, while 200 mg LCZ696 only reduced msPP; however, this may have been a consequence of the small sample size. The fact that LCZ696 reduced PP relative to placebo indicates that LCZ696 has the ability to reduce PP. The ability of LCZ696 to reduce PP reflects the fact that it reduces SBP better than DBP. LCZ696 outperforms 20 mg olmesartan in reducing PP, which may be caused by either its ability to reduce BP or LCZ696 dosage. Since PP is an independent predictor of cardiovascular events (myocardial infarction, congestive heart failure, and cardiovascular death) in hypertensive patients and normal people [25, 26], the finding that LCZ696 reduces PP better than 20 mg olmesartan suggests that this drug may provide hypertensive patients with greater protection from cardio-cerebrovascular diseases (*e.g.*, stroke and diastolic heart failure [27, 28]) than 20 mg olmesartan. Only a few clinical trials have compared the effectiveness LCZ696 with that of other dosages of olmesartan in reducing pulse pressure (PP), so large scale clinical studies are required to address this issue.

To summarize, LCZ696 was more effective in reducing blood pressure than placebo. Compared with 20 mg olmesartan, LCZ696 was more effective in reducing BP, indicating that LCZ696 is a promising agent; however, we cannot confirm that LCZ696 was more effective in reducing BP than other dosages of olmesartan.

Many clinical trials have reported that omapatrilat, which inhibits various proteases including neprilysin and angiotensin-converting enzyme (ACE), may increase the risk of angioedema when it is used to treat hypertension [10, 11]. By contrast, our meta-analysis of the nine eligible RCTs indicated that LCZ696 does not seem to increase the risk of angioedema when it is used to treat hypertension. This difference may reflect the fact that while LCZ696 consists of a neprilysin inhibitor and an ARB, omapatrilat simultaneously inhibits neprilysin and ACE [29]. Both neprilysin and ACE participate in the degradation of bradykinins [30, 31]. Thus, it may be that when neprilysin and ACE are inhibited at the same time, bradykinins accumulate, which in turn results in cough and angioedema [32]. Since LCZ696 contains an ARB rather than an angiotensin converting enzyme inhibitor (ACEI), ARB has no effect on bradykinin accumulation, thus reducing the risk of angioedema. However, since LCZ696 inhibits neprilysin, the risk of angioedema still theoretically exists. One RCT reported that peripheral edema occurred in a LCZ696 group [15]. Although there was no significant difference between the LCZ696 group and the olmesartan group (p=0.18), attention should be given to the risk of angioedema and peripheral edema. Future studies on the safety of LCZ696 for treating hypertension should assess the risk of angioedema and peripheral edema.

LCZ696 is a novel angiotensin receptor-neprilysin inhibitor, and was better at reducing blood pressure than olmesartan at a dose of 20mg or placebo. In addition, unlike omapatrilat, an angiotensin converting enzyme-neprilysin inhibitor, the administration of LCZ696 would not increase the risk of angioedema. So, we think LCZ696 as a promissing novel agent in treating hypertension.

A study of the effectiveness of LCZ696 in treating hypertension [33] showed that LCZ696 combined with amlodipine outperformed amlodipine alone in terms of reducing BP. Moreover, there was no significant difference between the two therapy strategies in terms of adverse events. However, this study did not assess whether LCZ696 combined with amlodipine outperforms LCZ696 alone in terms of reducing BP. Future studies on this issue are still wanted, especially since it has been shown that combining LCZ696 with HCTZ, amlodipine, or carvedilol is safe [34].

Two recent large clinical trials, namely, PARADIGM-HF [35] and PARAMOUNT [36], tested whether LCZ696 is useful for treating heart failure. These trials showed that compared with enalapril or valsartan, LCZ696 provides significantly greater benefits for heart failure patients. However, a number of unresolved questions remain. First, can taking LCZ696 as soon as hypertension emerges postpone the onset of heart failure? Second, does long-term treatment with LCZ696 weaken its performance in heart failure?

There is an article meta-analysis which included 6 randomized controlled trials (RCTs) on treating heart failure and hypertension with LCZ696, and evaluated the safety of LCZ696. But it does not distinguish patients with hypertension or heart failure, nor distinguish LCZ696 dose in the experimental groups. In order to illustrate the curative effect and safety of LCZ696 in the treatment of hypertension more accurately, we have just selected the RCTs on the treatment of patients with hypertension. This paper divides the RCTs into four subgroups according to the LCZ696 dosage the drug types of control group (placebo or 20mg olmesartan). And this article adds the latest RCTs and increases the sample size.

In the study by Zhao et al.[37], two drugs, including valsartan and olmesartan were used in ARB group, and the dose of olmesartan varied at 80mg, 160mg and 320mg in NCT00549770. The category and dose of drugs is likely to affect the effectiveness of reducing blood pressure. In this meta-analysis, olmesartan was only administered at a dose of 20mg, which can more objectively reflect the effectiveness of LCZ696 and olmesartan in reducing blood pressure. In this meta-analysis, the effectiveness and safety of LCZ696 and placebo in reducing blood pressure were compared, which contributes to the evaluation of the effectiveness and safety of LCZ696 in directly reducing blood pressure. Two RCTs, NCT01870739 and NCT01193101 enrolled in this paper, were not included in the study of Zhao et al.

### Limitations of this meta-analysis

This study also has some limitations. First, only nine RCTs were eligible for analysis. Second, most of the RCTs had a short follow-up duration, so studies with longer follow-up durations are needed. Third, some unpublished data from the website clinicaltrials.gov were included in this analysis.

Several questions require further investigation. First, compared with other anti-hypertensive agents, is LCZ696 better at preventing heart failure? Second, is the prognosis of long-term use of LCZ696 in terms of cardiovascular outcomes better than the prognosis of its short-term use? Additional clinical trials with long follow-up durations and large sample sizes are needed to address these issues.

## MATERIALS AND METHODS

### Search strategy

PubMed, EMBASE, EBSCO, the Cochrane database, and ClinicalTrials.gov were searched for relevant English language articles on clinical trials by using the search terms “LCZ696”, “LCZ-696”, “entresto”, “sacubitril-valsartan”, “sacubitril”, “endopeptidase”, “neutral endopeptidase”, and “neprilysin”. The last search date was July 20th, 2017.

### Selection criteria

The inclusion criteria were: ① the experimental subjects were diagnosed with hypertension; ② the clinical trial was a randomized controlled trial (RCT); ③ LCZ was given as a monotherapy for the duration of follow-up; ④ placebo or 20 mg olmesartan was used to treat the hypertensive patients in the control group; ⑤ the change in patient BP was recorded; and ⑥ the language of the paper was English.

The exclusion criteria were: ① the trial was not controlled or randomized; ② the hypertensive patients in the experimental group were treated with not only LCZ696 but also other antihypertensive drugs; and ③ the article with the same content was published repeatedly.

### Methodology and quality assessment

Two reviewers (Liwen Ye and Jian Wang) independently assessed the RCTs that met the eligibility criteria by using the Cochrane Collaboration bias risk tool. This tool examines whether the RCT involved proper random sequence generation, concealment of subject group allocation, blinding during outcome assessment, and complete recording and reporting of outcome data, and lacked other experimental biases [13]. This meta-analysis was reported according to the Preferred Reporting Items for Systematic Reviews and Meta-Analyses (PRISMA) Statement. Consequently, a flow diagram and the 27-item PRISMA checklist are included in this paper.

### Data extraction and management

All RCTs that met the eligibility criteria were included in this meta-analysis. Two researchers (Liwen Ye and Xixi Yang) extracted the data regarding patient characteristics and endpoints. To assess the ability of LCZ696 to control hypertension, the RCTs were divided into four groups depending on the dosage of LCZ696 and whether the control group received 20 mg olmesartan or placebo.

Various primary and secondary endpoints were used in the RCTs to assess the efficacy of LCZ696 for treating hypertension. The primary endpoints included changes relative to baseline in (I) mean sitting systolic blood pressure (msSBP), (II) mean ambulatory systolic blood pressure (maSBP), (III) mean sitting diastolic blood pressure (msDBP), (IV) mean ambulatory diastolic blood pressure (maDBP), (V) mean sitting pulse pressure (msPP), and (VI) mean ambulatory pulse pressure (maPP). These primary endpoint data were extracted in the form of mean±standard deviation (SD). In terms of mean ambulatory BP, the mean 24 hours ambulatory BP was selected, rather than mean daytime or nighttime ambulatory BP. (VII) The safety of LCZ696 involved was assessed on the basis of the adverse events (AEs), and (VIII) serious adverse events (SAEs), defined as death, arrhythmia, myocardial infarction, edema, malignancy, crebral infarction, subarachnoid haemorrhage and dyspnea.

Four RCTs tested LCZ696 at different doses (NCT00549770, NCT01193101, NCT01599104, and NCT01785472). Therefore, the experimental group was divided into different groups according to the dose of LCZ696. Three of the RCTs (NCT00549770, NCT01193101, NCT01281306 and NCT01692301) did not report the SD of the BP values in the published paper [12, 14, 15]. This information was extracted from ClinicalTrials.gov.

### Statistical analysis

This meta-analysis was performed by using Review Manager 5.3 statistical software, which is provided by the Cochrane Collaboration. The respective efficacy statistics for continuous and dichotomous outcomes were weighted mean difference (WMD) and risk ratio (RR) with 95% confidence intervals (CI).

In this meta-analysis, the Breslow-Day χ^2^-test (p-value) and the I^2^ statistic were used to test the heterogeneity of the RCTs. When p<0.1 and I^2^<25%, the RCT was classified as having low heterogeneity; when p<0.1 and 25%2<50%I^2^<50%, the RCT was classified as having moderate heterogeneity; and when p>0.1 and I^2^>50%, the RCT was classified as having high heterogeneity [16]. When p<0.1 and I^2^<50%, fixed effects Mantel-Haenzel model was used to analyze the data, whereas the random effects DerSimonian and Laird model was used when p>0.1 and I^2^>50%. A funnel plot and Egger's test were used to assess publication bias [17].

## CONCLUSION

LCZ696 at 200 mg or 400 mg was better at reducing most of BP parameters than 20 mg olmesartan or placebo. However, there was no significant difference between 200 mg LCZ696 and 20 mg olmesartan in reducing maPP. Compared with placebo or 20 mg olmesartan, 200 mg or 400 mg LCZ696 do not result in more adverse events in treating hypertension.

## References

[1] Kearney PM, Whelton M, Reynolds K, Muntner P, Whelton PK, He J (2005). Global burden of hypertension: analysis of worldwide data. Lancet.

[2] Pandit K, Mukhopadhyay P, Ghosh S, Chowdhury S (2011). Natriuretic peptides: diagnostic and therapeutic use. Indian J Endocrinol Metab.

[3] Sible AM, Nawarskas JJ, Alajajian D, Anderson JR (2016). Sacubitril/valsartan: a novel cardiovascular combination agent. Cardiol Rev.

[4] Volpe M, Carnovali M, Mastromarino V (2016). The natriuretic peptides system in the pathophysiology of heart failure: from molecular basis to treatment. Clin Sci.

[5] Volpe M (2014). Natriuretic peptides and cardio-renal disease. Int J Cardiol.

[6] Langenickel TH, Dole WP (2012). Angiotensin receptor-neprilysin inhibition with LCZ696: a novel approach for the treatment of heart failure. Drug Discov Today.

[7] Good JM, Peters M, Wilkins M, Jackson N, Oakley CM, Cleland JG (1995). Renal response to candoxatrilat in patients with heart failure. J Am Coll Cardiol.

[8] Cleland JG, Swedberg K (1998). Lack of efficacy of neutral endopeptidase inhibitor ecadotril in heart failure. The International Ecadotril Multi-centre Dose-ranging Study Investigators. Lancet.

[9] Bevan EG, Connell JM, Doyle J, Carmichael HA, Davies DL, Lorimer AR, McInnes GT (1992). Candoxatril, a neutral endopeptidase inhibitor: efficacy and tolerability in essential hypertension. J Hypertens.

[10] Kostis JB, Packer M, Black HR, Schmieder R, Henry D, Levy E (2004). Omapatrilat and enalapril in patients with hypertension: the Omapatrilat Cardiovascular Treatment vs. Enalapril (OCTAVE) trial. Am J Hypertens.

[11] Fryer RM, Segreti J, Banfor PN, Widomski DL, Backes BJ, Lin CW, Ballaron SJ, Cox BF, Trevillyan JM, Reinhart GA, von Geldern TW (2008). Effect of bradykinin metabolism inhibitors on evoked hypotension in rats: rank efficacy of enzymes associated with bradykinin-mediated angioedema. Br J Pharmacol.

[12] Kario K, Sun N, Chiang FT, Supasyndh O, Baek SH, Inubushi-Molessa A, Zhang Y, Gotou H, Lefkowitz M, Zhang J (2014). Efficacy and safety of LCZ696, a first-in-class angiotensin receptor neprilysin inhibitor, in Asian patients with hypertension: a randomized, double-blind, placebo-controlled study. Hypertension.

[13] Moher D, Liberati A, Tetzlaff J, Altman DG, PRISMA Group (2010). Preferred reporting items for systematic reviews and meta-analyses: the PRISMA statement. Int J Surg.

[14] Ruilope LM, Dukat A, Bohm M, Lacourciere Y, Gong J, Lefkowitz MP (2010). Blood-pressure reduction with LCZ696, a novel dual-acting inhibitor of the angiotensin II receptor and neprilysin: a randomised, double-blind, placebo-controlled, active comparator study. Lancet.

[15] Williams B, Cockcroft JR, Kario K, Zappe DH, Brunel PC, Wang Q, Guo W (2017). Effects of sacubitril/valsartan versus olmesartan on central hemodynamics in the elderly with systolic hypertension: the PARAMETER study. Hypertension.

[16] Higgins JP, Thompson SG, Deeks JJ, Altman DG (2003). Measuring inconsistency in meta-analyses. BMJ.

[17] Sterne JA, Egger M, Smith GD (2001). Systematic reviews in health care: investigating and dealing with publication and other biases in meta-analysis. BMJ.

[18] Izzo JL, Zappe DH, Jia Y, Hafeez K, Zhang J (2017). Efficacy and safety of crystalline valsartan/sacubitril (LCZ696) compared with placebo and combinations of free valsartan and sacubitril in patients with systolic hypertension: the RATIO study. J Cardiovasc Pharmacol.

[19] Borghi C, Rossi F, SIF Task Force; SIIA Task Force (2015). Role of the renin-angiotensin-aldosterone system and its pharmacological inhibitors in cardiovascular diseases: complex and critical issues. High Blood Press Cardiovasc Prev.

[20] Nishikimi T, Kuwahara K, Nakao K (2011). Current biochemistry, molecular biology, and clinical relevance of natriuretic peptides. J Cardiol.

[21] Standeven KF, Hess K, Carter AM, Rice GI, Cordell PA, Balmforth AJ, Lu B, Scott DJ, Turner AJ, Hooper NM, Grant PJ (2011). Neprilysin, obesity and the metabolic syndrome. Int J Obes (Lond).

[22] McMurray JJ, Packer M, Desai AS, Gong J, Lefkowitz MP, Rizkala AR, Rouleau J, Shi VC, Solomon SD, Swedberg K, Zile MR, PARADIGM-HF Committees and Investigators (2013). Dual angiotensin receptor and neprilysin inhibition as an alternative to angiotensin-converting enzyme inhibition in patients with chronic systolic heart failure: rationale for and design of the Prospective comparison of ARNI with ACEI to Determine Impact on Global Mortality and morbidity in Heart Failure trial (PARADIGM-HF). Eur J Heart Fail.

[23] Gu J, Noe A, Chandra P, Al-Fayoumi S, Ligueros-Saylan M, Sarangapani R, Maahs S, Ksander G, Rigel DF, Jeng AY, Lin TH, Zheng W, Dole WP (2010). Pharmacokinetics and pharmacodynamics of LCZ696, a novel dual-acting angiotensin receptor-neprilysin inhibitor (ARNi). J Clin Pharmacol.

[24] Wang TD, Tan RS, Lee HY, Ihm SH, Rhee MY, Tomlinson B, Pal P, Yang F, Hirschhorn E, Prescott MF, Hinder M, Langenickel TH (2017). Effects of sacubitril/valsartan (LCZ696) on natriuresis, diuresis, blood pressures, and NT-proBNP in salt-sensitive hypertension. Hypertension.

[25] Niiranen TJ, Rissanen H, Johansson JK, Jula AM (2014). Overall cardiovascular prognosis of isolated systolic hypertension, isolated diastolic hypertension and pulse pressure defined with home measurements: the Finn-home study. J Hypertens.

[26] Garcia-Palmieri MR, Crespo CJ, Mc Gee D, Sempos C, Smit E, Sorlie PD (2005). Wide pulse pressure is an independent predictor of cardiovascular mortality in Puerto Rican men. Nutr Metab Cardiovasc Dis.

[27] Safar ME (2001). Systolic blood pressure, pulse pressure and arterial stiffness as cardiovascular risk factors. Curr Opin Nephrol Hypertens.

[28] Haider AW, Larson MG, Franklin SS, Levy D, Framingham Heart Study (2003). Systolic blood pressure, diastolic blood pressure, and pulse pressure as predictors of risk for congestive heart failure in the Framingham Heart Study. Ann Intern Med.

[29] Ichiki T, Izumi R, Cataliotti A, Larsen AM, Sandberg SM, Burnett JC (2013). Endothelial permeability *in vitro* and *in vivo*: protective actions of ANP and omapatrilat in experimental atherosclerosis. Peptides.

[30] Kokkonen JO, Kuoppala A, Saarinen J, Lindstedt KA, Kovanen PT (1999). Kallidin- and bradykinin-degrading pathways in human heart: degradation of kallidin by aminopeptidase M-like activity and bradykinin by neutral endopeptidase. Circulation.

[31] Bayes-Genis A, Morant-Talamante N, Lupon J (2016). Neprilysin and natriuretic peptide regulation in heart failure. Curr Heart Fail Rep.

[32] Vleeming W, van Amsterdam JG, Stricker BH, de Wildt DJ, ACE inhibitor-induced angioedema (1998). Incidence, prevention and management. Drug Saf.

[33] Wang JG, Yukisada K, Sibulo A, Hafeez K, Jia Y, Zhang J (2017). Efficacy and safety of sacubitril/valsartan (LCZ696) add-on to amlodipine in Asian patients with systolic hypertension uncontrolled with amlodipine monotherapy. J Hypertens.

[34] Hsiao HL, Langenickel TH, Greeley M, Roberts J, Zhou W, Pal P, Rebello S, Rajman I, Sunkara G (2015). Pharmacokinetic drug-drug interaction assessment between LCZ696, an angiotensin receptor neprilysin inhibitor, and hydrochlorothiazide, amlodipine, or carvedilol. Clin Pharmacol Drug Dev.

[35] McMurray JJ, Packer M, Desai AS, Gong J, Lefkowitz MP, Rizkala AR, Rouleau JL, Shi VC, Solomon SD, Swedberg K, Zile MR, PARADIGM-HF Investigators and Committees (2014). Angiotensin-neprilysin inhibition versus enalapril in heart failure. N Engl J Med.

[36] Solomon SD, Zile M, Pieske B, Voors A, Shah A, Kraigher-Krainer E, Shi V, Bransford T, Takeuchi M, Gong J, Lefkowitz M, Packer M, JJ; McMurray, Prospective comparison of ARNI with ARB on Management Of heart failUre with preserved ejectioN fracTion (PARAMOUNT) Investigators (2012). The angiotensin receptor neprilysin inhibitor LCZ696 in heart failure with preserved ejection fraction: a phase 2 double-blind randomised controlled trial. Lancet.

[37] Zhao Y, Yu H, Zhao X, Ma R, Li N, Yu J (2017). The effects of LCZ696 in patients with hypertension compared with angiotensin receptor blockers: a meta-analysis of randomized controlled trials. J Cardiovasc Pharmacol Ther.

